# Anterior cervical discectomy and fusion may be more effective than anterior cervical corpectomy and fusion for the treatment of cervical spondylotic myelopathy

**DOI:** 10.1186/s12891-015-0490-9

**Published:** 2015-02-13

**Authors:** Li Guan, Yong Hai, Jin-Cai Yang, Li-Jin Zhou, Xiao-Long Chen

**Affiliations:** Department of Orthopedics, Beijing Chao-Yang Hospital, Capital Medical University, Gong Ti Nan Lu 8#, Chao-Yang District, Beijing, 100020 People’s Republic of China

**Keywords:** Anterior cervical corpectomy and fusion, Anterior cervical discectomy and fusion, Cervical spondylotic myelopathy, Meta-analysis

## Abstract

**Background:**

This meta-analysis explored the efficacy and safety of anterior cervical corpectomy and fusion (ACCF) comparing to anterior cervical discectomy and fusion (ACDF) in treating cervical spondylotic myelopathy (CSM) patients.

**Methods:**

Several electronic databases were searched combined with manually searching. Thirteen randomized controlled studies were enrolled with 1,062 CSM patients, including 468 patients and 594 patients in the in the ACCF and ACDF group, respectively. The meta-analysis was then performed using the STATA 12.0 software. Crude standard mean difference (SMD) or odds ratio (OR) with their 95% confidence intervals (CI) were calculated.

**Results:**

Our meta-analysis results revealed that CSM patients in ACDF group showed less blood loss than those in ACCF group (SMD = 1.21, 95% CI = 1.03 ~ 1.39, *P* < 0.001). The operation time of CSM patients in the ACDF group was also obviously shorter than those in ACCF group (SMD = 0.40, 95% CI = 0.23 ~ 0.57, *P* < 0.001). Furthermore, CSM patients in ACDF group had shorter hospital time than those in ACCF group (SMD = 0.45, 95% CI = 0.21 ~ 0.69, *P* < 0.001).

**Conclusion:**

Our findings provide empirical evidence that ACDF may be more effective than ACCF for CSM treatment.

## Background

As a progressive degenerative process, cervical spondylosis has major impacts on the cervical vertebral bodies and intervertebral discs [1]. Cervical spondylotic myelopathy (CSM), characterized by the compression of spinal cord, is a commonly disorder of progressive spinal cord, and the prevalence of which accounts for 10% ~ 15% of cervical spondylosis [2,3]. In a national cohort of eastern Asia, the incidence of CSM-induced hospitalization was 4.04 per 100,000 person every year, and CSM was reported to be associated with higher incidences in older and male patients [4]. CSM is the predominant reason of spinal cord injury and neurological dysfunction especially among industrialized countries, which may lead to disability as a life-long event, posing a great social and economic burden [4,5]. It has been highlighted that the compromise of spinal canal as well as the compression of spinal cord were the main pathology of CSM, and the etiology of CSM was suggested to be the age-related degenerative spondylosis [6,7]. At present, patients diagnosed with symptomatic CSM were often recommended to receive anterior cervical decompression and fusion for patients diagnosed with CSM [8,9].

ACDF is a surgical procedure focusing on the cervical spine through a small incision, and then removing the intervertebral disc, replaced by a small plug of bone or other graft substitute, which usually applied for the treating the compression of nerve root or spinal cord [10,11]. ACCF refers to a procedure removing part of the vertebra and adjacent intervertebral discs to allow for cervical spinal cord and nerves decompression. In the procedure, a bone graft, and sometimes a metal plate and screws, will be used to stabilize the spine [12,13]. In term of the clinical outcomes of the two surgical methods for CSM, it has been revealed that ACDF is more effective for CSM patients since ACDF was evidenced to significantly elevate the rates of fusion [14,15]. Additionally, ACCF has been confirmed to be beneficial for the treatment of cervical degenerative diseases, contributing to a direct neural structures decompression, immediate operated segments stabilization, solid fusion or restoration of cervical alignment; and consequently result in a short term follow-up of those patients [16-18]. However, there were higher induction of complications in the application of ACCF, including vertebral artery, dural tears and CSM leakage [19,20]. And ACDF may not be optimal for a higher risk of incomplete decompression, injury to the cord, limited visual exposure, and pseudarthrosis secondary to an increase in the number of fusion surfaces [19,21]. Furthermore, it was revealed that ACDF could result in greater improvements in cervical lordosis and segmental height as well as less blood loss than ACCF [13]. While ACCF was suggested to provide improved visualization over ACDF in the removal of the osteophytes and ossified [18]. Since the clinical outcomes between ACCF and ACDF were controversial, we conducted the current meta-analysis to compare the efficacy and safety between ACCF and ACDF in CSM treatment.

## Methods

### Eligible articles searching and selection

Related articles were searched in the following databases including: Web of Science (1945 ~ 2014),Cochrane (Issue 1, 2014), PubMed (1966 ~ 2014) and Chinese Biomedical Database (CBM, 1982 ~ 2014). Our study adopted the following MeSH terms and keywords: [“cervical spondylotic myelopathy” or “CSM”] and [“anterior cervical fusion” or “anterior cervical corpectomy with fusion” or “ACCF” or “anterior cervical discectomy with fusion” or “ACDF”]. Manual search was also conducted to seek other potential related articles based upon references identified in the retrieved articles.

The following criteria were used to determine eligibility for including studies: (1) study design must be randomized controlled study about the comparisons of the efficacy and safety between ACCF and ACDF in the treatment of CSM patients; (2) the study should had a mean follow-up of more than 6 months; (3) the study should offer complete data for assessment of clinical efficacy and the security of ACCF and ACDF; (4) the study must be Chinese or English document. Article that did not accord with the inclusive criteria were excluded. When authors published some related studies using the same study subjects, either the latest paper or the largest sample size article was included.

### Data extraction and quality assessment

**Figure 1:**
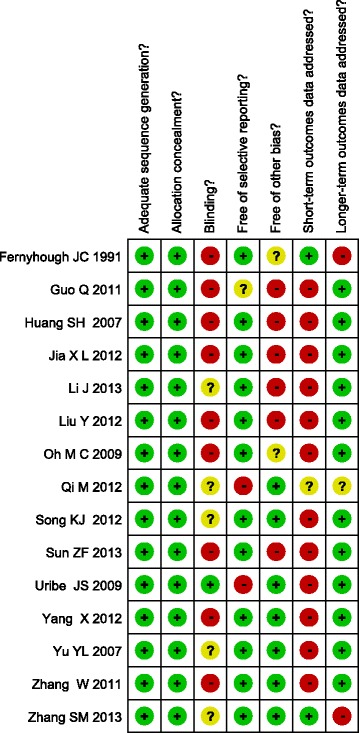
**The methodological quality of 13 included studies based on the revised Jadad score system.**

Initially, our search strategy identified 304 articles. We attentively checked the titles and abstracts and removed 150 articles. After systematically reviewing the remaining full texts, we excluded another 137 articles. Additionally, 3 studies were further excluded because of the deficiency of data integrity. Finally, 13 randomized controlled studies with 1,062 CSM patients, including 468 patients receiving ACCF and 594 patients undergoing ACDF, were eligible for the following statistical analysis [20-32]. Publication years of the included studies were between 2007 and 2013. Baseline characteristics and quality assessment of eligible studies were summarized in Table 1 and Figure 1. Two authors used to extract the following data from included studies: the first author, publication year, country, language of publication, race, study design, number of cases, age, duration of follow-up, clinical efficacy, operation time, hospital stay, blood loss during operation, the fusion rate, Japanese orthopedic association (JOA) score before and after operation, etc.

**Table 1:** **Baseline characteristics and methodological quality of all included studies**

**First author**	**Year**	**Country**	**Language**	**Journal**	**Case number**		**Gender** **(male/** **female)**		**Age** **(year)**		**Follow-** **up time** **(month)**	
					**ACCF**	**ACDF**	**ACCF**	**ACDF**	**ACCF**	**ACDF**	**ACCF**	**ACDF**
Zhang SM [31]	2013	China	Chinese	Chinese Journal of Bone Joint Injury	17	15	10/7	9/6	56.6 (52 ~ 68)	57.3 (50 ~ 64)	12	
Li J[22]	2013	China	English	Arch Orthop Trauma Surg	42	47	58/31		51.3 ± 6.5		79.6 (60 ~ 108)	
Sun ZF [28]	2013	China	Chinese	Chinese Journal of Bone Joint Injury	16	24	28/12		52.3 (35 ~ 70)		13 ~ 34	
Song KJ [20]	2012	Korea	English	Eur Spine J	15	25	11/4	19/6	54.1 ± 9.8	50.3 ± 7.5	72 ~ 171	61 ~ 132
Qi M [27]	2012	China	Chinese	Chinese Journal of Spine and Spinal Cord	94	124	51/43	69/55	54.4 ± 7.8	53.5 ± 8.5	-	42 (18 ~ 60)
Liu Y [26]	2012	China	English	Spine (Phila Pa 1976)	39	69	26/13	39/30	47.8 ± 6.4	46.1 ± 6.8	26.4 (12 ~ 37)	26.8 (12 ~ 39)
Jia XL [25]	2012	China	Chinese	Orthopedic Journal of China	36	31	21/15	17/14	48.8 ± 8.1	49.1 ± 7.7	25 ~ 61	
Zhang W [32]	2011	China	Chinese	Orthopedic Journal of China	69	87	92/64		55.1 ± 12.2	52.6 ± 11.4	27.3 ± 20	24.9 ± 24
Guo Q [23]	2011	China	English	Eur Spine J	24	43	13/11	24/19	55.2 ± 10.1	52.7 ± 9.4	37.3 ± 7.3	37.3 ± 7.2
Uribe JS [29]	2009	USA	English	Eur Spine J	38	42	21/17	21/21	50	46.2	26.4	27.6
Oh MC [21]	2009	Korea	English	Spine (Phila Pa 1976)	17	14	16/15		54.5 ± 11.6		12 ~ 63	
Yu YL [30]	2007	China	Chinese	Journal of Qiqihar Medical College	20	20	14/6	15/5	53.1 ± 8.9	52.8 ± 7.8	6	
Huang SH [24]	2007	China	Chinese	Practical Clinical Medicine	21	23	-	-	-	-	22 (12 ~ 63)	

*ACCF* anterior cervical corpectomy and fusion, *ACDF* anterior cervical discectomy and fusion.

Methodological quality was assessed individually by two observers through the revised Jadad score system [33]. The Jadad criterion is based on the following six aspects: (1) Adequate sequence generation? (2) Allocation concealment? (3) Blinding? (4) Incomplete outcome data addressed? (5) Free of selective reporting? (6) Free of other bias? And a score ≥ 3 indicates that the article has a good quality.

### Statistical analysis

The current Meta-analysis was conducted with Version 12.0 STATA statistical software (Stata Corporation, College Station, TX, USA). The efficacy and safety between ACCF and ACDF for the treatment of CSM was evaluated by calculating the standard mean difference (SMD), odds ratios (OR) and 95% confidence intervals (95% CI). The *Z* test was adopted to assess the statistical significance of the pooled ORs. Heterogeneity among studies was evaluated by the Cochran’s *Q*-statistic and *I*^*2*^ tests [34]. If the *Q*-test showed a *P* < 0.05 or the *I*^*2*^ test showed > 50%, which indicate significant heterogeneity, the random-effects model was used. Otherwise we used the fixed-effects model [35]. For the sake of evaluating the potential influence of each study on the overall results, we conducted a sensitivity analysis. In order to investigate the publication bias, both funnel plots and Egger’s linear regression test were performed [36]. All statistical tests were two-sided. A *P*-value < 0.05 showed a significance in statistical analysis.

## Results

### Quantitative data synthesis

**Figure 2:**
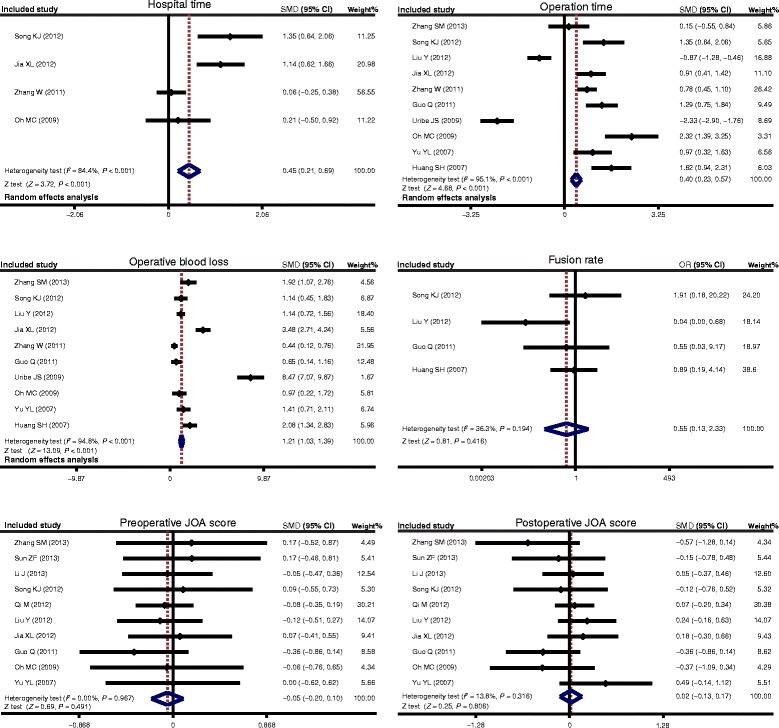
**Forest plots for the relationships based on hospital time,** **operation time,** **operative blood loss,** **fusion rate,** **preoperative JOA score and postoperative JOA score for the relationships of anterior cervical corpectomy and fusion with anterior cervical discectomy and fusion in patients with cervical spondylotic myelopathy.**

**Figure 3:**
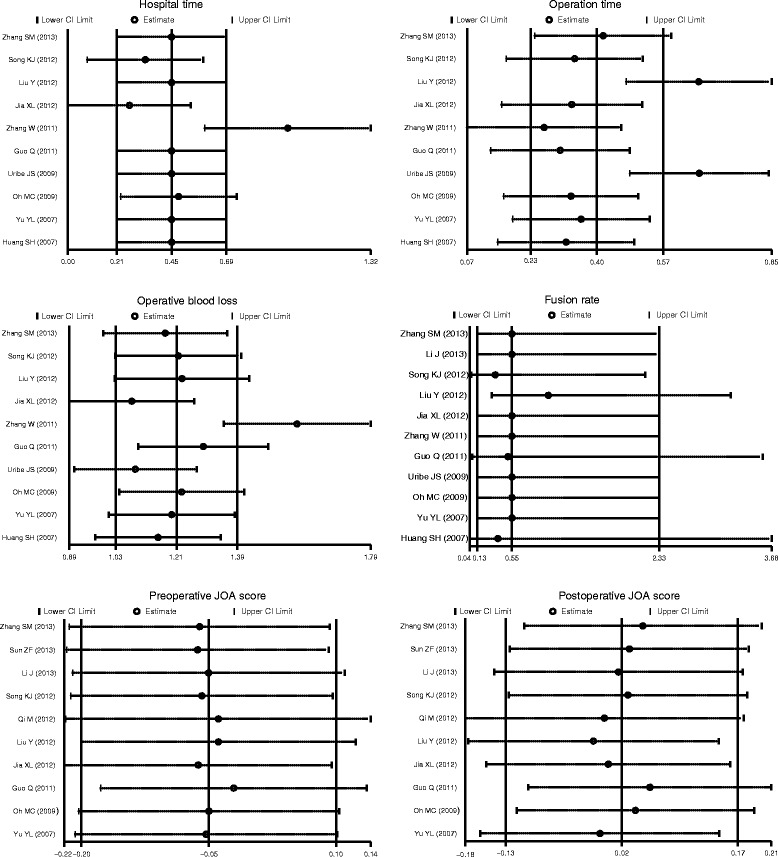
**Sensitivity analysis of the summary odds ratio coefficients for the relationships of anterior cervical corpectomy and fusion with anterior cervical discectomy and fusion in patients with cervical spondylotic myelopathy.**

**Figure 4:**
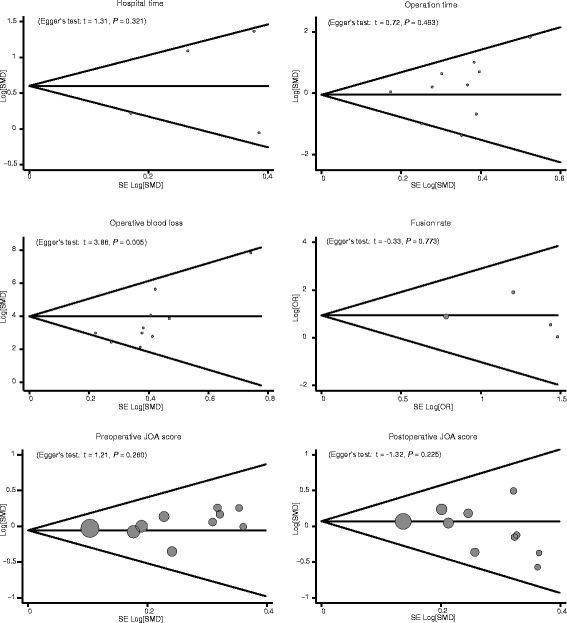
**Funnel plot of publication biases for the relationships of anterior cervical corpectomy and fusion with anterior cervical discectomy and fusion in patients with cervical spondylotic myelopathy.**

Totally ten articles were included for the comparison of operative blood loss and operation time in CSM patients between received ACDF treatment and treated with ACCF groups, these results indicate that CSM patients in treated with ACDF group showed less blood loss than those in received ACCF treatment group (SMD = 1.21, 95% CI = 1.03 ~ 1.39, *P* < 0.001). The operation time of ACDF was obviously shorter than that of ACCF (SMD = 0.40, 95% CI = 0.23 ~ 0.57, *P* < 0.001). In addition, there were only four studies enrolled comparing hospital time in CSM patients between the ACDF group and ACCF group, and CSM patients in the ACDF group had shorter hospital time than those in the ACCF group (SMD = 0.45, 95% CI = 0.21 ~ 0.69, *P* < 0.001). However, there was no presence of difference in the fusion rate (in four articles), preoperative JOA scores as well as postoperative JOA scores (in ten articles) in both groups (all *P* > 0.05) (Figure 2). A sensitivity analysis revealed each included study did not clearly influence the pooled ORs (Figure 3). Funnel plots suggested no existence of obvious asymmetry (Figure 4). No strong presence of publication bias was also shown by using Egger’s test (all *P >* 0.05).

## Discussion

Various different approaches have been applied to decompressive surgery of the cervical spine, such as multilevel discectomy, corpectomy, laminectomy with/without fusion, laminoplasty, and laminectomy [21,37]. Both approaches (anterior and posterior approaches) could contribute to the achievement of sufficient decompression of the spinal cord to improve clinical outcomes of CSM patients [38,39]. Anterior approach appears to be more suitable when the pathologies of anterior involve only 1 or 2 vertebral body levels, while if more than 2 levels usually proceed using an posterior approach clinically [40]. Additionally, cervical lordosis can be improved by both approaches, whereas anterior approaches present a relatively better overall correction for its higher probability of achieving release and distraction [41]. The present meta-analysis was mainly conducted to assess the efficacy and safety of anterior approaches (ACDF and ACCF) for managing CSM. Importantly, we discovered that the safety of ACDF was significantly superior as compared to ACCF with regard to the operation time, blood loss as well as hospitals time. Results in our study suggested that CSM patients received ACDF treatment showed less operative blood loss than those treated with ACCF. It has also been evidenced that as compared to ACCF, ACDF caused less blood loss as well as greater ameliorations in cervical lordosis and segmental height with better clinical outcomes [13]. Previous literature has described that the difference may probably due to the more invasive surgical approach of ACDF which was involved in removing a vertebral body [9,19]. We also found that the operation time of ACDF was obviously shorter than that of ACCF. Published studies suggested that ACCF was involved in the removal of about 15 ~ 19 mm of the anterior midline trough in the vertebral body down to the posterior longitudinal ligament, with elimination of the upper and lower adjacent discs; while ACDF was only associated with the excision of the affected intervertebral disc tissue [21,42]. So the ACCF was a complex procedure performed with technically more time consuming and challenging than ACDF. Our results was in line with a previous study confirming that ACCF had more operation time and blood loss compared to ACDF, and ACCF was inferior to ACDF in terms of segmental angle improvement and C2-7 angle improvement [23]. Furthermore, CSM patients in the ACDF group experienced shorter hospital time than those in the ACCF group. A potential explanation may be that patients receiving ACCF in the treatment of CSM may suffer from more serious spinal cord injury than patients undergoing ACDF, and ACDF was also suggested to be with less intraoperative blood loss; thus patients recover faster after ACDF surgery [23,43,44]. Multilevel ACDF may be related with high rates of fusion. The technique has well documented to be effective and safe for treating multilevel CSM resulting in less intraoperative blood loss, shorter operative times and shorter hospital stays for patients [21,38]. Consistent with our findings, Hwang et al. demonstrated that in multilevel cervical degenerative disc disease, multilevel discectomy and cage fusion with plate fixation is superior to corpectomy and struct graft fusion with plate fixation in terms of the absence of construct failures and donor site complications, along with shorter hospital stay [45]. However, we found no evident differences in the fusion rate, preoperative JOA scores and postoperative JOA scores in both groups, suggesting that there was no strong difference in the efficiency between ACCF and ACDF in the treatment of CSM.

Several limitations in this study should also be acknowledged. First, owing to the small sample size, there may be certain selection bias in our results. Second, despite the rigorous study design, the observer might be influenced by environmental factors, psychological factors, physical factors, theory and clinical experience, which may lead to bias in results. Third, original data from the selected studies was failed to be obtained in the present study that may limit further estimation of potential difference of the efficacy and safety between ACDF and ACCF in the treatment of CSM; thus limiting the clinical value of our study. Finally and importantly, ten in thirteen included articles were from China, which might affect the credibility and reliability of our results, and restricted the wide application of our findings.

## Conclusions

In conclusion, our meta-analysis reveals no significant difference in efficacy comparison between ACDF and ACCF, but the safety of ACDF was superior to ACCF with respect to the operation time, blood loss as well as hospital time. Thus, ACDF may be a safer alternative to ACCF for CSM patients, and may significantly result in the early rehabilitation for CSM patients. However, owing to the limitations of the current study, high-quality clinical studies with larger sample sizes are still needed to confirm our results.

## Abbreviations

CSM: Cervical spondylotic myelopathy
ACCF: Anterior cervical corpectomy and fusion
ACDF: Anterior cervical discectomy and fusion
JOA: Japanese orthopedic association
SMD: Standard mean difference
OR: Odds ratios
95% CI: 95% confidence intervals
